# *trans*-Dichloridobis[dicyclo­hex­yl(4-isopropyl­phen­yl)phosphane-κ*P*]platinum(II) acetone monosolvate

**DOI:** 10.1107/S1600536811051841

**Published:** 2011-12-07

**Authors:** Bubele Vuba, Alfred Muller

**Affiliations:** a Research Centre for Synthesis and Catalysis, Department of Chemistry, University of Johannesburg, PO Box 524, Auckland Park, Johannesburg 2006, South Africa

## Abstract

The title compound, [PtCl_2_(C_21_H_33_P)_2_]·C_3_H_6_O, crystallizes with an accompanying acetone solvent mol­ecule. The metal atom shows a distorted square-planar coordination environment, with a P—Pt—P angle of 172.41 (3)° as the most prominent feature. Both isopropyl fragments were treated as disordered over two conformations with occupancy ratios of 0.55 (2):0.45 (2) and 0.58 (2):0.42 (2). The solvent mol­ecule was also disordered over two orientations in a 1:1 ratio. The crystal studied was a non-merohedral twin with a twin component of 32.4%.

## Related literature

For background to our investigation of the steric and electronic effects of group 15 ligands, see Roodt *et al.* (2003 ▶); Muller *et al.* (2006 ▶, 2008 ▶). Examples of the packing disorder observed in Vaska-type complexes of Rh, Ir, Pd and Pt are given by Chen *et al.* (1991 ▶), Kuwabara & Bau (1994 ▶), Otto *et al.* (2000 ▶) and Otto (2001 ▶), respectively. For examples of Pt complexes with phospho­rus ligands in a *trans* orientation, see: Otto & Roodt (1997 ▶); Johansson *et al.* (2000 ▶) and for examples of Pt complexes with phospho­rus ligands in a *cis* orientation, see: Otto & Muller (2001 ▶), Otto & Johansson (2001 ▶). For the analogous Rh complex containing a dicyclo­hex­yl(4- isopropyl­phen­yl)phosphane ligand, see: Makhoba *et al.* (2011 ▶). For a description of the Cambridge Structural Database, see: (Allen, 2002 ▶). For background to cone angles, see: Tolman (1977 ▶). The twinned crystal was indexing using the *CELL_NOW* program (Bruker, 2008 ▶).
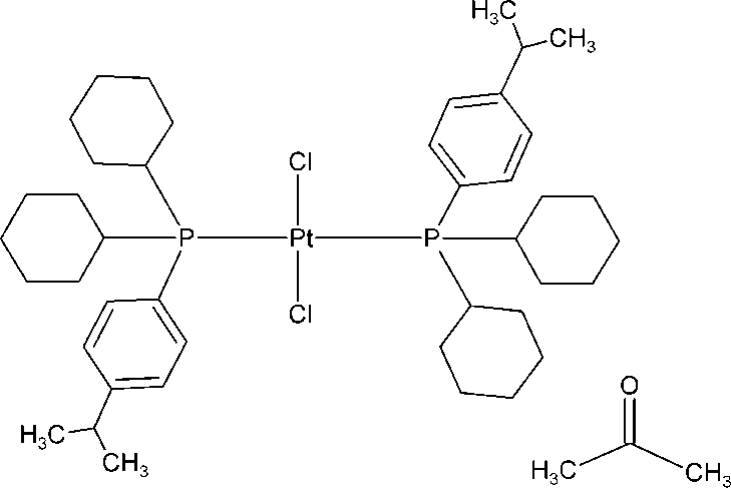


## Experimental

### 

#### Crystal data

[PtCl_2_(C_21_H_33_P)_2_]·C_3_H_6_O
*M* *_r_* = 956.96
Triclinic, 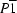

*a* = 10.407 (2) Å
*b* = 15.075 (3) Å
*c* = 15.766 (3) Å
α = 88.81 (3)°
β = 88.33 (3)°
γ = 74.17 (3)°
*V* = 2378.5 (8) Å^3^
*Z* = 2
Cu *K*α radiation
μ = 7.40 mm^−1^
*T* = 293 K
0.13 × 0.13 × 0.13 mm

#### Data collection

Bruker APEX DUO 4K CCD diffractometer
Absorption correction: multi-scan (TWINABS; Bruker, 2008 ▶) *T*_min_ = 0.446, *T*_max_ = 0.446
7557 measured reflections
7557 independent reflections
7055 reflections with *I* > 2σ(*I*)

#### Refinement

*R*[*F* ^2^ > 2σ(*F* ^2^)] = 0.026
*wR*(*F* ^2^) = 0.067
*S* = 1.11
7557 reflections
557 parameters
218 restraints
H-atom parameters constrained
Δρ_max_ = 0.72 e Å^−3^
Δρ_min_ = −0.75 e Å^−3^

### 

Data collection: *APEX2* (Bruker, 2011 ▶); cell refinement: *SAINT* (Bruker, 2008 ▶); data reduction: *SAINT* and *XPREP* (Bruker, 2008 ▶); program(s) used to solve structure: *SIR97* (Altomare *et al.*, 1999 ▶); program(s) used to refine structure: *SHELXL97* (Sheldrick, 2008 ▶); molecular graphics: *DIAMOND* (Brandenburg & Putz, 2005 ▶); software used to prepare material for publication: *WinGX* (Farrugia, 1999 ▶).

## Supplementary Material

Crystal structure: contains datablock(s) global, I. DOI: 10.1107/S1600536811051841/cv5210sup1.cif

Structure factors: contains datablock(s) I. DOI: 10.1107/S1600536811051841/cv5210Isup2.hkl

Additional supplementary materials:  crystallographic information; 3D view; checkCIF report

## Figures and Tables

**Fig. 1.**
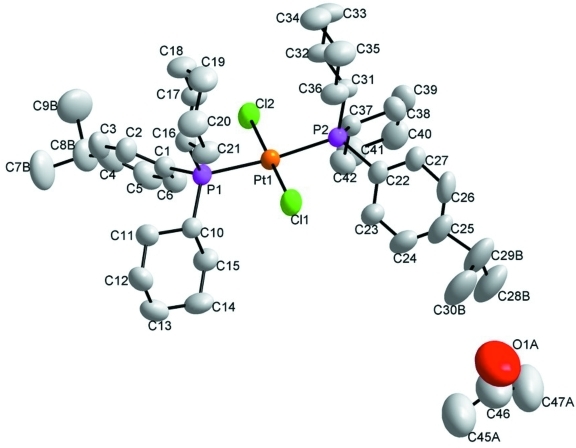
A view of the title compound showing the numbering scheme of atoms and 50% probability displacement ellipsoids. Hydrogen atoms and the minor components of disordered parts are omitted for clarity.

## supplementary crystallographic information

### Comment

Dihalo-bisphosphane complexes of platinum(II) are well documented in the literature. These complexes form part of a class of symmetrical square-planar complexes that usually crystallize with the metal atom on a crystallographic inversion center, thus imposing a disordered packing arrangement (see Otto, 2001; Otto *et al.*, 2000; Chen *et al.*,1991, Kuwabara & Bau, 1994 for examples on Rh, Ir, Pd and Pt, respectively). Very often the Pt complexes display a *trans* geometry (Otto & Roodt, 1997; Johansson *et al.*, 2000), but some number with a *cis* geometry have also been reported (Otto & Muller, 2001; Otto & Johansson, 2001). Pt(II) complexes, along with the Vaska-type complexes, are useful model complexes and provide several probing methods, *e.g.* NMR and IR, to investigate the steric and electronic effects of novel group 15 ligands (Roodt *et al.*, 2003; Muller *et al.*, 2006; Muller *et al.*, 2008). Reported here is the *trans*-[PtCl_2_{PCy_2_(4-C_3_H_7_—C_6_H_4_)}_2_] complex as a part of this ongoing study.

The title compound (Fig. 1) shows a packing arrangement of molecules lying in general positions in the unit cell, and thus no crystallographic symmetry imposed on the metal center as is usually observed with these complexes. The coordination environment of the Pt shows slight distortions from the ideal square-planar geometry. This deformation is most prominently observed in the P_1_—Pt_1_—P_2_ angle of 172.41 (3)°, whereas the Cl_1_—Pt_1_—Cl_2_ is almost linear at 178.94 (4)°. The metal complex is accompanied by an acetone solvate that had to be treated for disorder. Additionally the isopropyl moieties also showed large ellipsoid displacement parameters and were subsequently treated to individual disorder refinements.

An adaptation of the well known Tolman cone angle model (Tolman, 1977) was used for the determination of the phosphorus ligand bulkiness. Instead of using a CPK model, the actual geometry from the crystal structure was taken to determine an 'effective cone angle' (Otto *et al.* 2001). In addition the Pt—P distance was adjusted to 2.28 Å (the distance used by Tolman) to exclude deviations that the Pt—P bond may cause when comparing the steric values. Two almost similar cone angles of 165° and 166° were obtained for P_1_and P_2_ respectively, and compares reasonably to those obtained for the analogous Rh complex of this phosphorus ligand (Makhoba *et al.*, 2011).

### Experimental

Dichloro(1,5-cyclooctadiene)platinum(II), [PtCl_2_(COD)], and dicyclohexyl(4- isopropylphenyl)phosphane were purchased from Sigma-Aldrich and were used without purification. A solution of the phosphane (35 mg, 0.11 mmol) in acetone (5 ml) was added drop wise to a solution of [PtCl_2_ (COD)] (20 mg, 0.05 mmol) also in acetone (5 ml) while stirring at room temperature. This solution was evaporated, resulting in a yellow precipitate that was redissolved in acetone (10 ml). Slow evaporation of the solvent yielded crystals suitable for a single-crystal X-ray study.

### Refinement

All hydrogen atoms were positioned in geometrically idealized positions with C—H = 1.00 Å (methine), 0.99 Å (methylene), 0.98 Å (methyl) and 0.95 Å (aromatic). All hydrogen atoms were allowed to ride on their parent atoms with *U*_iso_(H) = 1.2*U*_eq_, except for the methyl where *U*_iso_(H) = 1.5*U*_eq_ was utilized. The initial positions of methyl hydrogen atoms were located from a Fourier difference map and refined as fixed rotor. Disorder refinement models were applied to both of the isopropyl fragments as well as the acetone solvate molecule. Several geometrical restraints (*DFIX*, DANG and FLAT) were applied. Values for *DFIX* and DANG parameters were obtained from averages of data mining searches from the Cambridge Structural Database (Allen, 2002; CSD ver. 5.32, August 2011 update). Ellipsoid displacement (SIMU and DELU) restraints were also applied to the disordered moieties. The occupation parameters of the two disordered isopropyls and the acetone were linked to free variables so that the two sites associated with each disorder would add to unity. Final occupancy ratios of 0.55:0.45 (2), 0.58:0.42 (2), 0.50:0.50 (3) were obtained. All restraints were applied with default standard deviations. Initial CheckCIF evaluation indicated possible non-merohedral twinning, and the data was subsequently treated using *CELL_NOW* (Bruker, 2008) to obtain orientation matrix of the two components. The raw data was then integrated as two components resulting in a HKLF5 format file, which greatly improved refinement parameters and yielded the refined composition of the twinned domains in a 32.4:67.6 ratio. The highest residual electron density 0.72 e.Å^-3^ was located 0.97 Å from Pt1, and the deepest hole of -0.75 e.Å^-3^ is 0.87 Å from Pt1. Both represent no physical meaning.

### Crystal data

**Table:**

[PtCl_2_(C_21_H_33_P)_2_]·C_3_H_6_O	*Z* = 2
*M**_r_* = 956.96	*F*(000) = 984
Triclinic, *P*1	*D*_x_ = 1.336 Mg m^−^^3^
Hall symbol: -P 1	Cu *K*α radiation, λ = 1.54178 Å
*a* = 10.407 (2) Å	Cell parameters from 9391 reflections
*b* = 15.075 (3) Å	θ = 6.4–63.6°
*c* = 15.766 (3) Å	µ = 7.40 mm^−^^1^
α = 88.81 (3)°	*T* = 293 K
β = 88.33 (3)°	Cubic, yellow
γ = 74.17 (3)°	0.13 × 0.13 × 0.13 mm
*V* = 2378.5 (8) Å^3^	

### Data collection

**Table:**

Bruker APEX DUO 4K CCD diffractometer	7557 measured reflections
Radiation source: Incoatec IµS microfocus X-ray source	7557 independent reflections
Incoatec Quazar Multilayer Mirror	7055 reflections with *I* > 2σ(*I*)
Detector resolution: 8.4 pixels mm^-1^	θ_max_ = 63.7°, θ_min_ = 5.6°
φ and ω scans	*h* = −12→12
Absorption correction: multi-scan (TWINABS; Bruker, 2008)	*k* = −17→17
*T*_min_ = 0.446, *T*_max_ = 0.446	*l* = 0→17

### Refinement

**Table:**

Refinement on *F*^2^	Primary atom site location: structure-invariant direct methods
Least-squares matrix: full	Secondary atom site location: difference Fourier map
*R*[*F*^2^ > 2σ(*F*^2^)] = 0.026	Hydrogen site location: inferred from neighbouring sites
*wR*(*F*^2^) = 0.067	H-atom parameters constrained
*S* = 1.11	*w* = 1/[σ^2^(*F*_o_^2^) + (0.0238*P*)^2^ + 1.6789*P*] where *P* = (*F*_o_^2^ + 2*F*_c_^2^)/3
7557 reflections	(Δ/σ)_max_ = 0.002
557 parameters	Δρ_max_ = 0.72 e Å^−^^3^
218 restraints	Δρ_min_ = −0.75 e Å^−^^3^

### Special details

**Table:**

Experimental. The intensity data was collected on a Bruker Apex DUO 4 K CCD diffractometer using an exposure time of 10 s/frame. A total of 5967 frames were collected with a frame width of 1° covering up to θ = 63.73° with 96.4% completeness accomplished.Analytical data: ^31^P {H} NMR (CDCl_3_, 160 MHz): δ = 21.29 (t, ^1^*J*_Pt—P_ = 2506 Hz, 2P)
Geometry. All e.s.d.'s (except the e.s.d. in the dihedral angle between two l.s. planes) are estimated using the full covariance matrix. The cell e.s.d.'s are taken into account individually in the estimation of e.s.d.'s in distances, angles and torsion angles; correlations between e.s.d.'s in cell parameters are only used when they are defined by crystal symmetry. An approximate (isotropic) treatment of cell e.s.d.'s is used for estimating e.s.d.'s involving l.s. planes.
Refinement. Refinement of *F*^2^ against ALL reflections. The weighted *R*-factor *wR* and goodness of fit *S* are based on *F*^2^, conventional *R*-factors *R* are based on *F*, with *F* set to zero for negative *F*^2^. The threshold expression of *F*^2^ > σ(*F*^2^) is used only for calculating *R*-factors(gt) *etc*. and is not relevant to the choice of reflections for refinement. *R*-factors based on *F*^2^ are statistically about twice as large as those based on *F*, and *R*- factors based on ALL data will be even larger.

### Fractional atomic coordinates and isotropic or equivalent isotropic displacement parameters (Å^2^)

**Table:**

	*x*	*y*	*z*	*U*_iso_*/*U*_eq_	Occ. (<1)
Pt1	0.037592 (14)	0.994590 (13)	0.748013 (11)	0.04399 (7)	
P1	0.01993 (9)	1.14101 (6)	0.68968 (6)	0.0418 (2)	
P2	0.02439 (10)	0.85413 (6)	0.80698 (6)	0.0432 (2)	
Cl1	0.03139 (13)	0.94136 (7)	0.61250 (7)	0.0659 (3)	
Cl2	0.03981 (14)	1.04956 (7)	0.88342 (7)	0.0660 (3)	
C1	0.0814 (4)	1.2182 (3)	0.7558 (3)	0.0494 (9)	
C2	0.0177 (5)	1.3110 (3)	0.7643 (3)	0.0656 (12)	
H2	−0.0607	1.3373	0.7356	0.079*	
C3	0.0723 (7)	1.3656 (4)	0.8165 (4)	0.0832 (17)	
H3	0.0278	1.4277	0.8224	0.1*	
C4	0.1908 (7)	1.3294 (5)	0.8595 (3)	0.0879 (18)	
C5	0.2539 (6)	1.2381 (5)	0.8483 (3)	0.0815 (16)	
H5	0.3343	1.2124	0.8751	0.098*	
C6	0.2015 (5)	1.1830 (4)	0.7983 (3)	0.0636 (12)	
H6	0.2471	1.121	0.7927	0.076*	
C7A	0.3262 (18)	1.4409 (18)	0.876 (2)	0.097 (6)	0.45 (2)
H7A1	0.3576	1.4763	0.9162	0.146*	0.45 (2)
H7A2	0.2972	1.4782	0.826	0.146*	0.45 (2)
H7A3	0.3973	1.3878	0.8602	0.146*	0.45 (2)
C8A	0.210 (2)	1.4100 (14)	0.9143 (9)	0.086 (6)	0.45 (2)
H8A	0.1288	1.4613	0.916	0.103*	0.45 (2)
C9A	0.239 (3)	1.3646 (16)	1.0032 (13)	0.118 (8)	0.45 (2)
H9A1	0.323	1.3176	1.0011	0.177*	0.45 (2)
H9A2	0.1691	1.3375	1.0202	0.177*	0.45 (2)
H9A3	0.2442	1.4105	1.0433	0.177*	0.45 (2)
C7B	0.268 (3)	1.4679 (13)	0.8831 (17)	0.136 (8)	0.55 (2)
H7B1	0.3167	1.4604	0.83	0.205*	0.55 (2)
H7B2	0.3076	1.5006	0.9216	0.205*	0.55 (2)
H7B3	0.1768	1.5021	0.874	0.205*	0.55 (2)
C8B	0.2710 (15)	1.3744 (10)	0.9204 (9)	0.083 (4)	0.55 (2)
H8B	0.3572	1.3359	0.9399	0.099*	0.55 (2)
C9B	0.161 (3)	1.421 (2)	0.9913 (17)	0.176 (11)	0.55 (2)
H9B1	0.2036	1.4437	1.036	0.264*	0.55 (2)
H9B2	0.1201	1.3753	1.0142	0.264*	0.55 (2)
H9B3	0.0947	1.4705	0.9664	0.264*	0.55 (2)
C10	0.1063 (4)	1.1447 (3)	0.5869 (3)	0.0458 (9)	
H10	0.0679	1.1107	0.5468	0.055*	
C11	0.0853 (4)	1.2419 (3)	0.5505 (3)	0.0586 (11)	
H11A	−0.0096	1.2707	0.5449	0.07*	
H11B	0.1199	1.2784	0.5893	0.07*	
C12	0.1548 (5)	1.2412 (4)	0.4643 (3)	0.0662 (12)	
H12A	0.1131	1.2109	0.4238	0.079*	
H12B	0.1438	1.3042	0.4447	0.079*	
C13	0.3016 (5)	1.1922 (4)	0.4674 (3)	0.0741 (14)	
H13A	0.3457	1.2268	0.5023	0.089*	
H13B	0.3408	1.1892	0.4106	0.089*	
C14	0.3238 (5)	1.0952 (4)	0.5039 (4)	0.0832 (16)	
H14A	0.4189	1.0668	0.5085	0.1*	
H14B	0.2887	1.0587	0.4654	0.1*	
C15	0.2562 (4)	1.0950 (4)	0.5911 (3)	0.0653 (12)	
H15A	0.2978	1.1257	0.6313	0.078*	
H15B	0.2679	1.032	0.6106	0.078*	
C16	−0.1595 (4)	1.1997 (3)	0.6715 (3)	0.0484 (9)	
H16	−0.1668	1.2643	0.6566	0.058*	
C17	−0.2442 (4)	1.1980 (4)	0.7524 (3)	0.0628 (12)	
H17A	−0.2135	1.23	0.797	0.075*	
H17B	−0.2323	1.1345	0.771	0.075*	
C18	−0.3934 (5)	1.2436 (4)	0.7382 (4)	0.0773 (15)	
H18A	−0.4439	1.2381	0.7898	0.093*	
H18B	−0.4066	1.3087	0.7257	0.093*	
C19	−0.4455 (5)	1.2000 (4)	0.6665 (4)	0.0807 (16)	
H19A	−0.5385	1.2322	0.6576	0.097*	
H19B	−0.44	1.1362	0.6808	0.097*	
C20	−0.3643 (5)	1.2044 (4)	0.5858 (4)	0.0804 (16)	
H20A	−0.3757	1.2684	0.5693	0.096*	
H20B	−0.3967	1.1744	0.5405	0.096*	
C21	−0.2151 (4)	1.1573 (4)	0.5983 (3)	0.0660 (13)	
H21A	−0.2029	1.0921	0.6099	0.079*	
H21B	−0.1656	1.1633	0.5464	0.079*	
C22	0.0841 (4)	0.7531 (3)	0.7397 (3)	0.0533 (10)	
C23	0.1963 (5)	0.7467 (3)	0.6873 (3)	0.0678 (12)	
H23	0.2379	0.7941	0.686	0.081*	
C24	0.2460 (6)	0.6698 (4)	0.6371 (4)	0.0872 (17)	
H24	0.3212	0.6667	0.6028	0.105*	
C25	0.1880 (7)	0.5978 (4)	0.6362 (4)	0.0860 (17)	
C26	0.0768 (7)	0.6035 (3)	0.6886 (4)	0.0805 (17)	
H26	0.0364	0.5555	0.6898	0.097*	
C27	0.0243 (5)	0.6806 (3)	0.7398 (3)	0.0609 (11)	
H27	−0.051	0.6836	0.774	0.073*	
C28A	0.306 (3)	0.4329 (13)	0.6221 (17)	0.120 (9)	0.42 (2)
H28A	0.2328	0.4201	0.6539	0.18*	0.42 (2)
H28B	0.3733	0.4385	0.6604	0.18*	0.42 (2)
H28C	0.3431	0.3835	0.5837	0.18*	0.42 (2)
C29A	0.257 (3)	0.5213 (13)	0.5729 (13)	0.104 (8)	0.42 (2)
H29A	0.3268	0.537	0.5377	0.124*	0.42 (2)
C30A	0.145 (3)	0.504 (2)	0.521 (2)	0.157 (12)	0.42 (2)
H30A	0.1826	0.4622	0.476	0.236*	0.42 (2)
H30B	0.0921	0.5609	0.4978	0.236*	0.42 (2)
H30C	0.0892	0.4767	0.5572	0.236*	0.42 (2)
C28B	0.369 (2)	0.459 (2)	0.5965 (19)	0.180 (12)	0.58 (2)
H28D	0.3917	0.4001	0.569	0.269*	0.58 (2)
H28E	0.3865	0.4496	0.656	0.269*	0.58 (2)
H28F	0.423	0.496	0.5721	0.269*	0.58 (2)
C29B	0.224 (2)	0.5068 (12)	0.5845 (11)	0.118 (8)	0.58 (2)
H29B	0.164	0.4675	0.5966	0.141*	0.58 (2)
C30B	0.230 (3)	0.5352 (14)	0.4902 (10)	0.165 (11)	0.58 (2)
H30D	0.3077	0.5565	0.4796	0.248*	0.58 (2)
H30E	0.1514	0.5837	0.4772	0.248*	0.58 (2)
H30F	0.2343	0.483	0.4553	0.248*	0.58 (2)
C31	−0.1547 (4)	0.8613 (3)	0.8346 (3)	0.0529 (10)	
H31	−0.1604	0.7995	0.8517	0.063*	
C32	−0.2056 (5)	0.9263 (4)	0.9089 (3)	0.0707 (14)	
H32A	−0.1953	0.9869	0.8945	0.085*	
H32B	−0.1528	0.9033	0.9584	0.085*	
C33	−0.3531 (5)	0.9340 (5)	0.9297 (4)	0.0915 (19)	
H33A	−0.3621	0.8743	0.9487	0.11*	
H33B	−0.3836	0.977	0.9758	0.11*	
C34	−0.4399 (5)	0.9666 (5)	0.8535 (4)	0.0893 (17)	
H34A	−0.5317	0.9682	0.8681	0.107*	
H34B	−0.4373	1.0285	0.8373	0.107*	
C35	−0.3911 (5)	0.9028 (5)	0.7804 (4)	0.0872 (17)	
H35A	−0.4443	0.9263	0.7312	0.105*	
H35B	−0.403	0.8426	0.7948	0.105*	
C36	−0.2425 (4)	0.8926 (4)	0.7578 (3)	0.0669 (12)	
H36A	−0.2136	0.848	0.7129	0.08*	
H36B	−0.2321	0.9513	0.737	0.08*	
C37	0.1175 (4)	0.8193 (3)	0.9054 (3)	0.0499 (9)	
H37	0.0799	0.8676	0.9469	0.06*	
C38	0.1019 (5)	0.7284 (3)	0.9450 (3)	0.0626 (11)	
H38A	0.0079	0.7338	0.9561	0.075*	
H38B	0.1356	0.6788	0.9051	0.075*	
C39	0.1774 (6)	0.7053 (4)	1.0273 (3)	0.0748 (14)	
H39A	0.136	0.7509	1.0695	0.09*	
H39B	0.1712	0.6457	1.0484	0.09*	
C40	0.3235 (6)	0.7030 (4)	1.0157 (4)	0.0810 (16)	
H40A	0.3678	0.6525	0.9789	0.097*	
H40B	0.3663	0.6922	1.0702	0.097*	
C41	0.3392 (6)	0.7933 (4)	0.9772 (4)	0.0868 (16)	
H41A	0.4333	0.7884	0.9673	0.104*	
H41B	0.3036	0.843	1.0167	0.104*	
C42	0.2655 (5)	0.8157 (4)	0.8929 (3)	0.0690 (13)	
H42A	0.2731	0.8747	0.871	0.083*	
H42B	0.3066	0.769	0.8516	0.083*	
C46	0.3293 (9)	0.4367 (8)	0.2435 (7)	0.159 (3)	
C47A	0.408 (3)	0.3606 (18)	0.297 (2)	0.236 (17)	0.50 (3)
H47A	0.4584	0.3845	0.3363	0.354*	0.50 (3)
H47B	0.4677	0.3157	0.2617	0.354*	0.50 (3)
H47C	0.3485	0.3321	0.3279	0.354*	0.50 (3)
C45A	0.379 (4)	0.5040 (19)	0.1990 (19)	0.193 (12)	0.50 (3)
H45A	0.3119	0.5385	0.1612	0.289*	0.50 (3)
H45B	0.4577	0.4739	0.1668	0.289*	0.50 (3)
H45C	0.3997	0.5449	0.2388	0.289*	0.50 (3)
O1A	0.2100 (18)	0.438 (3)	0.234 (2)	0.287 (17)	0.50 (3)
C47B	0.349 (4)	0.3403 (15)	0.267 (3)	0.246 (17)	0.50 (3)
H47D	0.4393	0.3144	0.2844	0.368*	0.50 (3)
H47E	0.3312	0.3071	0.2197	0.368*	0.50 (3)
H47F	0.2888	0.3358	0.3136	0.368*	0.50 (3)
C45B	0.443 (3)	0.439 (3)	0.183 (2)	0.239 (16)	0.50 (3)
H45D	0.4117	0.4826	0.1385	0.358*	0.50 (3)
H45E	0.48	0.3787	0.1599	0.358*	0.50 (3)
H45F	0.5113	0.4563	0.2136	0.358*	0.50 (3)
O1B	0.254 (4)	0.5038 (19)	0.273 (2)	0.35 (2)	0.50 (3)

### Atomic displacement parameters (Å^2^)

**Table:**

	*U*^11^	*U*^22^	*U*^33^	*U*^12^	*U*^13^	*U*^23^
Pt1	0.05352 (10)	0.04342 (10)	0.03564 (10)	−0.01379 (8)	−0.00328 (8)	−0.00393 (6)
P1	0.0444 (5)	0.0402 (5)	0.0407 (5)	−0.0105 (4)	−0.0071 (4)	−0.0027 (4)
P2	0.0507 (5)	0.0397 (5)	0.0386 (5)	−0.0111 (4)	0.0006 (4)	−0.0036 (4)
Cl1	0.1075 (8)	0.0549 (6)	0.0398 (6)	−0.0287 (6)	−0.0075 (5)	−0.0074 (4)
Cl2	0.1064 (8)	0.0555 (6)	0.0386 (6)	−0.0255 (6)	−0.0045 (5)	−0.0083 (4)
C1	0.058 (2)	0.052 (2)	0.042 (2)	−0.0203 (18)	−0.0018 (17)	−0.0065 (17)
C2	0.085 (3)	0.055 (3)	0.061 (3)	−0.024 (2)	−0.003 (2)	−0.011 (2)
C3	0.128 (5)	0.063 (3)	0.071 (4)	−0.049 (3)	0.012 (3)	−0.016 (3)
C4	0.128 (5)	0.112 (5)	0.054 (3)	−0.081 (4)	−0.007 (3)	−0.010 (3)
C5	0.088 (4)	0.112 (5)	0.065 (3)	−0.059 (3)	−0.020 (3)	−0.004 (3)
C6	0.063 (3)	0.072 (3)	0.060 (3)	−0.023 (2)	−0.017 (2)	−0.001 (2)
C7A	0.074 (9)	0.101 (15)	0.131 (13)	−0.048 (9)	0.006 (9)	−0.044 (11)
C8A	0.101 (12)	0.103 (13)	0.075 (9)	−0.060 (10)	−0.011 (8)	−0.026 (8)
C9A	0.17 (2)	0.136 (18)	0.073 (10)	−0.077 (14)	−0.043 (11)	−0.020 (10)
C7B	0.22 (2)	0.094 (12)	0.129 (14)	−0.097 (16)	−0.061 (15)	0.004 (11)
C8B	0.088 (9)	0.073 (8)	0.093 (9)	−0.030 (7)	−0.032 (6)	−0.009 (6)
C9B	0.22 (2)	0.22 (3)	0.125 (16)	−0.132 (17)	0.046 (14)	−0.116 (16)
C10	0.0449 (19)	0.047 (2)	0.045 (2)	−0.0120 (16)	−0.0055 (16)	−0.0039 (17)
C11	0.061 (2)	0.060 (3)	0.052 (3)	−0.013 (2)	0.0007 (19)	0.003 (2)
C12	0.078 (3)	0.075 (3)	0.051 (3)	−0.029 (2)	−0.003 (2)	0.006 (2)
C13	0.070 (3)	0.100 (4)	0.059 (3)	−0.036 (3)	0.008 (2)	−0.005 (3)
C14	0.060 (3)	0.092 (4)	0.085 (4)	−0.002 (3)	0.018 (3)	−0.010 (3)
C15	0.051 (2)	0.068 (3)	0.070 (3)	−0.005 (2)	−0.002 (2)	0.002 (2)
C16	0.046 (2)	0.048 (2)	0.050 (2)	−0.0098 (17)	−0.0083 (17)	0.0025 (17)
C17	0.055 (2)	0.078 (3)	0.057 (3)	−0.020 (2)	0.004 (2)	−0.012 (2)
C18	0.053 (2)	0.095 (4)	0.080 (4)	−0.013 (2)	0.010 (2)	−0.014 (3)
C19	0.047 (2)	0.101 (4)	0.094 (4)	−0.019 (3)	−0.011 (2)	−0.004 (3)
C20	0.055 (3)	0.107 (4)	0.077 (4)	−0.017 (3)	−0.022 (2)	−0.005 (3)
C21	0.054 (2)	0.089 (4)	0.053 (3)	−0.015 (2)	−0.011 (2)	−0.015 (2)
C22	0.065 (2)	0.045 (2)	0.046 (2)	−0.0091 (19)	−0.0069 (19)	−0.0033 (18)
C23	0.072 (3)	0.059 (3)	0.067 (3)	−0.010 (2)	0.013 (2)	−0.010 (2)
C24	0.095 (4)	0.078 (4)	0.071 (4)	0.006 (3)	0.020 (3)	−0.012 (3)
C25	0.117 (5)	0.056 (3)	0.065 (4)	0.011 (3)	−0.004 (3)	−0.015 (3)
C26	0.123 (5)	0.041 (3)	0.077 (4)	−0.018 (3)	−0.018 (3)	−0.008 (2)
C27	0.079 (3)	0.047 (2)	0.054 (3)	−0.011 (2)	−0.005 (2)	−0.006 (2)
C28A	0.18 (2)	0.038 (8)	0.116 (16)	0.013 (11)	0.015 (13)	−0.020 (8)
C29A	0.168 (17)	0.043 (8)	0.083 (15)	0.002 (11)	0.013 (11)	−0.032 (7)
C30A	0.20 (2)	0.118 (19)	0.13 (2)	0.006 (15)	−0.030 (16)	−0.080 (16)
C28B	0.175 (15)	0.14 (2)	0.160 (19)	0.069 (15)	−0.010 (15)	−0.053 (15)
C29B	0.144 (11)	0.094 (14)	0.093 (10)	0.003 (10)	0.008 (9)	−0.028 (9)
C30B	0.25 (3)	0.120 (14)	0.078 (9)	0.031 (14)	0.013 (11)	−0.039 (8)
C31	0.052 (2)	0.060 (3)	0.050 (3)	−0.0199 (19)	0.0091 (18)	−0.0065 (19)
C32	0.060 (3)	0.096 (4)	0.055 (3)	−0.019 (3)	0.012 (2)	−0.025 (3)
C33	0.068 (3)	0.126 (5)	0.077 (4)	−0.022 (3)	0.026 (3)	−0.024 (4)
C34	0.055 (3)	0.105 (5)	0.100 (5)	−0.010 (3)	0.015 (3)	−0.011 (4)
C35	0.062 (3)	0.116 (5)	0.085 (4)	−0.024 (3)	−0.006 (3)	−0.015 (3)
C36	0.057 (2)	0.087 (4)	0.053 (3)	−0.013 (2)	−0.004 (2)	−0.009 (2)
C37	0.060 (2)	0.049 (2)	0.041 (2)	−0.0148 (18)	−0.0027 (17)	−0.0010 (17)
C38	0.078 (3)	0.063 (3)	0.050 (3)	−0.024 (2)	−0.010 (2)	0.007 (2)
C39	0.102 (4)	0.068 (3)	0.051 (3)	−0.017 (3)	−0.010 (3)	0.013 (2)
C40	0.086 (4)	0.081 (4)	0.066 (3)	−0.004 (3)	−0.026 (3)	0.007 (3)
C41	0.077 (3)	0.100 (4)	0.086 (4)	−0.027 (3)	−0.028 (3)	0.011 (3)
C42	0.062 (3)	0.080 (3)	0.068 (3)	−0.023 (2)	−0.011 (2)	0.013 (3)
C46	0.112 (5)	0.151 (7)	0.200 (10)	−0.018 (6)	0.020 (7)	0.019 (7)
C47A	0.24 (3)	0.116 (18)	0.37 (4)	−0.063 (18)	−0.07 (3)	0.051 (19)
C45A	0.22 (3)	0.128 (19)	0.25 (3)	−0.078 (19)	0.00 (2)	0.003 (16)
O1A	0.137 (11)	0.31 (4)	0.42 (4)	−0.081 (16)	−0.014 (17)	0.08 (3)
C47B	0.19 (3)	0.174 (14)	0.37 (5)	−0.06 (2)	−0.02 (3)	0.07 (2)
C45B	0.17 (2)	0.28 (4)	0.25 (3)	−0.05 (2)	0.053 (18)	0.05 (3)
O1B	0.30 (4)	0.22 (2)	0.43 (4)	0.09 (2)	0.12 (3)	0.00 (2)

### Geometric parameters (Å, °)

**Table:**

Pt1—Cl1	2.3065 (12)	C24—C25	1.379 (9)
Pt1—Cl2	2.3090 (12)	C24—H24	0.93
Pt1—P2	2.3315 (11)	C25—C26	1.387 (9)
Pt1—P1	2.3358 (11)	C25—C29A	1.547 (16)
P1—C1	1.829 (4)	C25—C29B	1.561 (15)
P1—C10	1.838 (4)	C26—C27	1.404 (7)
P1—C16	1.863 (4)	C26—H26	0.93
P2—C22	1.828 (4)	C27—H27	0.93
P2—C37	1.844 (4)	C28A—C29A	1.500 (18)
P2—C31	1.877 (4)	C28A—H28A	0.96
C1—C2	1.383 (6)	C28A—H28B	0.96
C1—C6	1.402 (6)	C28A—H28C	0.96
C2—C3	1.410 (7)	C29A—C30A	1.525 (19)
C2—H2	0.93	C29A—H29A	0.98
C3—C4	1.394 (9)	C30A—H30A	0.96
C3—H3	0.93	C30A—H30B	0.96
C4—C5	1.367 (9)	C30A—H30C	0.96
C4—C8A	1.566 (14)	C28B—C29B	1.509 (18)
C4—C8B	1.570 (12)	C28B—H28D	0.96
C5—C6	1.382 (6)	C28B—H28E	0.96
C5—H5	0.93	C28B—H28F	0.96
C6—H6	0.93	C29B—C30B	1.542 (18)
C7A—C8A	1.515 (16)	C29B—H29B	0.98
C7A—H7A1	0.96	C30B—H30D	0.96
C7A—H7A2	0.96	C30B—H30E	0.96
C7A—H7A3	0.96	C30B—H30F	0.96
C8A—C9A	1.547 (17)	C31—C36	1.525 (6)
C8A—H8A	0.98	C31—C32	1.530 (6)
C9A—H9A1	0.96	C31—H31	0.98
C9A—H9A2	0.96	C32—C33	1.533 (7)
C9A—H9A3	0.96	C32—H32A	0.97
C7B—C8B	1.507 (16)	C32—H32B	0.97
C7B—H7B1	0.96	C33—C34	1.517 (9)
C7B—H7B2	0.96	C33—H33A	0.97
C7B—H7B3	0.96	C33—H33B	0.97
C8B—C9B	1.600 (17)	C34—C35	1.503 (8)
C8B—H8B	0.98	C34—H34A	0.97
C9B—H9B1	0.96	C34—H34B	0.97
C9B—H9B2	0.96	C35—C36	1.544 (7)
C9B—H9B3	0.96	C35—H35A	0.97
C10—C11	1.525 (6)	C35—H35B	0.97
C10—C15	1.538 (6)	C36—H36A	0.97
C10—H10	0.98	C36—H36B	0.97
C11—C12	1.519 (6)	C37—C42	1.533 (6)
C11—H11A	0.97	C37—C38	1.540 (6)
C11—H11B	0.97	C37—H37	0.98
C12—C13	1.506 (7)	C38—C39	1.522 (6)
C12—H12A	0.97	C38—H38A	0.97
C12—H12B	0.97	C38—H38B	0.97
C13—C14	1.519 (8)	C39—C40	1.517 (8)
C13—H13A	0.97	C39—H39A	0.97
C13—H13B	0.97	C39—H39B	0.97
C14—C15	1.526 (7)	C40—C41	1.526 (8)
C14—H14A	0.97	C40—H40A	0.97
C14—H14B	0.97	C40—H40B	0.97
C15—H15A	0.97	C41—C42	1.539 (7)
C15—H15B	0.97	C41—H41A	0.97
C16—C21	1.532 (6)	C41—H41B	0.97
C16—C17	1.533 (6)	C42—H42A	0.97
C16—H16	0.98	C42—H42B	0.97
C17—C18	1.539 (7)	C46—O1B	1.193 (14)
C17—H17A	0.97	C46—O1A	1.249 (14)
C17—H17B	0.97	C46—C45A	1.420 (15)
C18—C19	1.505 (7)	C46—C47B	1.455 (16)
C18—H18A	0.97	C46—C47A	1.479 (16)
C18—H18B	0.97	C46—C45B	1.503 (16)
C19—C20	1.516 (8)	C47A—H47A	0.96
C19—H19A	0.97	C47A—H47B	0.96
C19—H19B	0.97	C47A—H47C	0.96
C20—C21	1.537 (6)	C45A—H45A	0.96
C20—H20A	0.97	C45A—H45B	0.96
C20—H20B	0.97	C45A—H45C	0.96
C21—H21A	0.97	C47B—H47D	0.96
C21—H21B	0.97	C47B—H47E	0.96
C22—C23	1.393 (7)	C47B—H47F	0.96
C22—C27	1.397 (6)	C45B—H45D	0.96
C23—C24	1.388 (7)	C45B—H45E	0.96
C23—H23	0.93	C45B—H45F	0.96
			
Cl1—Pt1—Cl2	178.94 (4)	C25—C24—H24	118.8
Cl1—Pt1—P2	91.33 (4)	C23—C24—H24	118.8
Cl2—Pt1—P2	88.98 (4)	C24—C25—C26	117.7 (5)
Cl1—Pt1—P1	88.54 (4)	C24—C25—C29A	113.6 (13)
Cl2—Pt1—P1	91.02 (4)	C26—C25—C29A	128.7 (13)
P2—Pt1—P1	172.41 (3)	C24—C25—C29B	131.6 (10)
C1—P1—C10	103.70 (19)	C26—C25—C29B	110.7 (10)
C1—P1—C16	106.24 (19)	C25—C26—C27	120.9 (5)
C10—P1—C16	105.70 (18)	C25—C26—H26	119.6
C1—P1—Pt1	115.25 (14)	C27—C26—H26	119.6
C10—P1—Pt1	116.19 (13)	C22—C27—C26	120.6 (5)
C16—P1—Pt1	108.92 (14)	C22—C27—H27	119.7
C22—P2—C37	103.27 (19)	C26—C27—H27	119.7
C22—P2—C31	105.9 (2)	C28A—C29A—C30A	103 (3)
C37—P2—C31	106.2 (2)	C28A—C29A—C25	108.0 (17)
C22—P2—Pt1	116.34 (15)	C30A—C29A—C25	105.8 (17)
C37—P2—Pt1	115.10 (14)	C28A—C29A—H29A	113.1
C31—P2—Pt1	109.17 (15)	C30A—C29A—H29A	113.1
C2—C1—C6	117.6 (4)	C25—C29A—H29A	113.1
C2—C1—P1	123.4 (3)	C29B—C28B—H28D	109.5
C6—C1—P1	118.9 (3)	C29B—C28B—H28E	109.5
C1—C2—C3	119.7 (5)	H28D—C28B—H28E	109.5
C1—C2—H2	120.2	C29B—C28B—H28F	109.5
C3—C2—H2	120.2	H28D—C28B—H28F	109.5
C4—C3—C2	122.1 (5)	H28E—C28B—H28F	109.5
C4—C3—H3	118.9	C28B—C29B—C30B	99 (3)
C2—C3—H3	118.9	C28B—C29B—C25	108.1 (16)
C5—C4—C3	117.2 (5)	C30B—C29B—C25	106.5 (14)
C5—C4—C8A	136.9 (11)	C28B—C29B—H29B	113.9
C3—C4—C8A	105.8 (10)	C30B—C29B—H29B	113.9
C5—C4—C8B	110.8 (8)	C25—C29B—H29B	113.9
C3—C4—C8B	132.0 (8)	C29B—C30B—H30D	109.5
C4—C5—C6	121.7 (5)	C29B—C30B—H30E	109.5
C4—C5—H5	119.1	H30D—C30B—H30E	109.5
C6—C5—H5	119.1	C29B—C30B—H30F	109.5
C5—C6—C1	121.7 (5)	H30D—C30B—H30F	109.5
C5—C6—H6	119.2	H30E—C30B—H30F	109.5
C1—C6—H6	119.2	C36—C31—C32	110.3 (4)
C7A—C8A—C9A	113 (3)	C36—C31—P2	110.8 (3)
C7A—C8A—C4	107.9 (16)	C32—C31—P2	111.0 (3)
C9A—C8A—C4	102.9 (15)	C36—C31—H31	108.2
C7A—C8A—H8A	110.8	C32—C31—H31	108.2
C9A—C8A—H8A	110.8	P2—C31—H31	108.2
C4—C8A—H8A	110.8	C31—C32—C33	110.9 (4)
C8B—C7B—H7B1	109.5	C31—C32—H32A	109.5
C8B—C7B—H7B2	109.5	C33—C32—H32A	109.5
H7B1—C7B—H7B2	109.5	C31—C32—H32B	109.5
C8B—C7B—H7B3	109.5	C33—C32—H32B	109.5
H7B1—C7B—H7B3	109.5	H32A—C32—H32B	108
H7B2—C7B—H7B3	109.5	C34—C33—C32	111.7 (5)
C7B—C8B—C4	106.7 (14)	C34—C33—H33A	109.3
C7B—C8B—C9B	91 (2)	C32—C33—H33A	109.3
C4—C8B—C9B	103.0 (12)	C34—C33—H33B	109.3
C7B—C8B—H8B	117.4	C32—C33—H33B	109.3
C4—C8B—H8B	117.4	H33A—C33—H33B	107.9
C9B—C8B—H8B	117.4	C35—C34—C33	110.1 (5)
C8B—C9B—H9B1	109.5	C35—C34—H34A	109.6
C8B—C9B—H9B2	109.5	C33—C34—H34A	109.6
H9B1—C9B—H9B2	109.5	C35—C34—H34B	109.6
C8B—C9B—H9B3	109.5	C33—C34—H34B	109.6
H9B1—C9B—H9B3	109.5	H34A—C34—H34B	108.2
H9B2—C9B—H9B3	109.5	C34—C35—C36	112.0 (4)
C11—C10—C15	110.5 (4)	C34—C35—H35A	109.2
C11—C10—P1	113.5 (3)	C36—C35—H35A	109.2
C15—C10—P1	111.8 (3)	C34—C35—H35B	109.2
C11—C10—H10	106.9	C36—C35—H35B	109.2
C15—C10—H10	106.9	H35A—C35—H35B	107.9
P1—C10—H10	106.9	C31—C36—C35	111.5 (4)
C12—C11—C10	111.5 (4)	C31—C36—H36A	109.3
C12—C11—H11A	109.3	C35—C36—H36A	109.3
C10—C11—H11A	109.3	C31—C36—H36B	109.3
C12—C11—H11B	109.3	C35—C36—H36B	109.3
C10—C11—H11B	109.3	H36A—C36—H36B	108
H11A—C11—H11B	108	C42—C37—C38	110.8 (4)
C13—C12—C11	112.0 (4)	C42—C37—P2	111.7 (3)
C13—C12—H12A	109.2	C38—C37—P2	113.9 (3)
C11—C12—H12A	109.2	C42—C37—H37	106.7
C13—C12—H12B	109.2	C38—C37—H37	106.7
C11—C12—H12B	109.2	P2—C37—H37	106.7
H12A—C12—H12B	107.9	C39—C38—C37	111.2 (4)
C12—C13—C14	111.0 (4)	C39—C38—H38A	109.4
C12—C13—H13A	109.4	C37—C38—H38A	109.4
C14—C13—H13A	109.4	C39—C38—H38B	109.4
C12—C13—H13B	109.4	C37—C38—H38B	109.4
C14—C13—H13B	109.4	H38A—C38—H38B	108
H13A—C13—H13B	108	C40—C39—C38	112.1 (4)
C13—C14—C15	112.0 (4)	C40—C39—H39A	109.2
C13—C14—H14A	109.2	C38—C39—H39A	109.2
C15—C14—H14A	109.2	C40—C39—H39B	109.2
C13—C14—H14B	109.2	C38—C39—H39B	109.2
C15—C14—H14B	109.2	H39A—C39—H39B	107.9
H14A—C14—H14B	107.9	C39—C40—C41	111.4 (4)
C14—C15—C10	110.5 (4)	C39—C40—H40A	109.4
C14—C15—H15A	109.6	C41—C40—H40A	109.4
C10—C15—H15A	109.6	C39—C40—H40B	109.4
C14—C15—H15B	109.6	C41—C40—H40B	109.4
C10—C15—H15B	109.6	H40A—C40—H40B	108
H15A—C15—H15B	108.1	C40—C41—C42	110.9 (5)
C21—C16—C17	109.8 (4)	C40—C41—H41A	109.5
C21—C16—P1	112.3 (3)	C42—C41—H41A	109.5
C17—C16—P1	110.9 (3)	C40—C41—H41B	109.5
C21—C16—H16	107.9	C42—C41—H41B	109.5
C17—C16—H16	107.9	H41A—C41—H41B	108.1
P1—C16—H16	107.9	C37—C42—C41	110.8 (4)
C16—C17—C18	111.9 (4)	C37—C42—H42A	109.5
C16—C17—H17A	109.2	C41—C42—H42A	109.5
C18—C17—H17A	109.2	C37—C42—H42B	109.5
C16—C17—H17B	109.2	C41—C42—H42B	109.5
C18—C17—H17B	109.2	H42A—C42—H42B	108.1
H17A—C17—H17B	107.9	O1B—C46—O1A	68.0 (18)
C19—C18—C17	111.8 (4)	O1B—C46—C45A	81.9 (18)
C19—C18—H18A	109.2	O1A—C46—C45A	118.3 (18)
C17—C18—H18A	109.2	O1B—C46—C47B	129 (2)
C19—C18—H18B	109.2	O1A—C46—C47B	85.2 (17)
C17—C18—H18B	109.2	C45A—C46—C47B	148 (2)
H18A—C18—H18B	107.9	O1B—C46—C47A	122 (2)
C18—C19—C20	110.1 (4)	O1A—C46—C47A	115.4 (17)
C18—C19—H19A	109.6	C45A—C46—C47A	126.2 (18)
C20—C19—H19A	109.6	O1B—C46—C45B	124 (2)
C18—C19—H19B	109.6	O1A—C46—C45B	134 (2)
C20—C19—H19B	109.6	C47B—C46—C45B	105.7 (19)
H19A—C19—H19B	108.2	C47A—C46—C45B	95 (2)
C19—C20—C21	111.3 (4)	C46—C47A—H47A	109.5
C19—C20—H20A	109.4	C46—C47A—H47B	109.5
C21—C20—H20A	109.4	C46—C47A—H47C	109.5
C19—C20—H20B	109.4	C46—C45A—H45A	109.5
C21—C20—H20B	109.4	C46—C45A—H45B	109.5
H20A—C20—H20B	108	C46—C45A—H45C	109.5
C16—C21—C20	111.7 (4)	C46—C47B—H47D	109.5
C16—C21—H21A	109.3	C46—C47B—H47E	109.5
C20—C21—H21A	109.3	H47D—C47B—H47E	109.5
C16—C21—H21B	109.3	C46—C47B—H47F	109.5
C20—C21—H21B	109.3	H47D—C47B—H47F	109.5
H21A—C21—H21B	107.9	H47E—C47B—H47F	109.5
C23—C22—C27	118.2 (4)	C46—C45B—H45D	109.5
C23—C22—P2	118.7 (3)	C46—C45B—H45E	109.5
C27—C22—P2	123.1 (3)	H45D—C45B—H45E	109.5
C24—C23—C22	120.1 (5)	C46—C45B—H45F	109.5
C24—C23—H23	120	H45D—C45B—H45F	109.5
C22—C23—H23	120	H45E—C45B—H45F	109.5
C25—C24—C23	122.5 (5)		
			
Cl1—Pt1—P1—C1	−157.29 (15)	C16—C17—C18—C19	55.9 (6)
Cl2—Pt1—P1—C1	23.67 (15)	C17—C18—C19—C20	−56.7 (7)
Cl1—Pt1—P1—C10	−35.71 (14)	C18—C19—C20—C21	57.1 (7)
Cl2—Pt1—P1—C10	145.25 (14)	C17—C16—C21—C20	54.3 (6)
Cl1—Pt1—P1—C16	83.46 (15)	P1—C16—C21—C20	178.2 (4)
Cl2—Pt1—P1—C16	−95.57 (15)	C19—C20—C21—C16	−56.9 (6)
Cl1—Pt1—P2—C22	24.79 (16)	C37—P2—C22—C23	−89.4 (4)
Cl2—Pt1—P2—C22	−156.22 (16)	C31—P2—C22—C23	159.2 (4)
Cl1—Pt1—P2—C37	145.77 (15)	Pt1—P2—C22—C23	37.7 (4)
Cl2—Pt1—P2—C37	−35.24 (15)	C37—P2—C22—C27	89.2 (4)
Cl1—Pt1—P2—C31	−94.97 (15)	C31—P2—C22—C27	−22.3 (4)
Cl2—Pt1—P2—C31	84.02 (15)	Pt1—P2—C22—C27	−143.8 (3)
C10—P1—C1—C2	92.3 (4)	C27—C22—C23—C24	−0.2 (7)
C16—P1—C1—C2	−18.9 (4)	P2—C22—C23—C24	178.4 (4)
Pt1—P1—C1—C2	−139.6 (3)	C22—C23—C24—C25	0.4 (9)
C10—P1—C1—C6	−85.6 (4)	C23—C24—C25—C26	−0.7 (9)
C16—P1—C1—C6	163.3 (4)	C23—C24—C25—C29A	177.7 (10)
Pt1—P1—C1—C6	42.5 (4)	C23—C24—C25—C29B	178.5 (10)
C6—C1—C2—C3	−1.9 (7)	C24—C25—C26—C27	0.9 (8)
P1—C1—C2—C3	−179.9 (4)	C29A—C25—C26—C27	−177.2 (11)
C1—C2—C3—C4	1.0 (8)	C29B—C25—C26—C27	−178.5 (8)
C2—C3—C4—C5	0.8 (9)	C23—C22—C27—C26	0.4 (7)
C2—C3—C4—C8A	−176.2 (7)	P2—C22—C27—C26	−178.2 (4)
C2—C3—C4—C8B	−179.9 (8)	C25—C26—C27—C22	−0.7 (8)
C3—C4—C5—C6	−1.5 (9)	C24—C25—C29A—C28A	120 (2)
C8A—C4—C5—C6	174.1 (10)	C26—C25—C29A—C28A	−62 (3)
C8B—C4—C5—C6	179.0 (7)	C29B—C25—C29A—C28A	−58 (4)
C4—C5—C6—C1	0.6 (9)	C24—C25—C29A—C30A	−130 (2)
C2—C1—C6—C5	1.2 (7)	C26—C25—C29A—C30A	48 (3)
P1—C1—C6—C5	179.2 (4)	C29B—C25—C29A—C30A	52 (4)
C5—C4—C8A—C7A	75 (2)	C24—C25—C29B—C28B	53 (2)
C3—C4—C8A—C7A	−109 (2)	C26—C25—C29B—C28B	−128 (2)
C8B—C4—C8A—C7A	65 (3)	C29A—C25—C29B—C28B	55 (4)
C5—C4—C8A—C9A	−45 (2)	C24—C25—C29B—C30B	−53 (2)
C3—C4—C8A—C9A	130.7 (19)	C26—C25—C29B—C30B	126.1 (19)
C8B—C4—C8A—C9A	−56 (2)	C29A—C25—C29B—C30B	−51 (4)
C5—C4—C8B—C7B	138.5 (17)	C22—P2—C31—C36	−72.3 (4)
C3—C4—C8B—C7B	−41 (2)	C37—P2—C31—C36	178.3 (3)
C8A—C4—C8B—C7B	−49 (2)	Pt1—P2—C31—C36	53.7 (3)
C5—C4—C8B—C9B	−126.5 (18)	C22—P2—C31—C32	164.8 (4)
C3—C4—C8B—C9B	54 (2)	C37—P2—C31—C32	55.4 (4)
C8A—C4—C8B—C9B	46 (2)	Pt1—P2—C31—C32	−69.2 (4)
C1—P1—C10—C11	−55.2 (3)	C36—C31—C32—C33	55.3 (6)
C16—P1—C10—C11	56.4 (3)	P2—C31—C32—C33	178.5 (4)
Pt1—P1—C10—C11	177.3 (2)	C31—C32—C33—C34	−57.2 (7)
C1—P1—C10—C15	70.6 (3)	C32—C33—C34—C35	56.9 (7)
C16—P1—C10—C15	−177.8 (3)	C33—C34—C35—C36	−55.7 (7)
Pt1—P1—C10—C15	−56.9 (3)	C32—C31—C36—C35	−54.3 (6)
C15—C10—C11—C12	55.4 (5)	P2—C31—C36—C35	−177.6 (4)
P1—C10—C11—C12	−178.2 (3)	C34—C35—C36—C31	55.4 (7)
C10—C11—C12—C13	−55.6 (5)	C22—P2—C37—C42	71.8 (4)
C11—C12—C13—C14	54.8 (6)	C31—P2—C37—C42	−177.0 (3)
C12—C13—C14—C15	−55.3 (6)	Pt1—P2—C37—C42	−56.1 (4)
C13—C14—C15—C10	55.7 (6)	C22—P2—C37—C38	−54.7 (4)
C11—C10—C15—C14	−55.3 (5)	C31—P2—C37—C38	56.6 (4)
P1—C10—C15—C14	177.3 (4)	Pt1—P2—C37—C38	177.5 (3)
C1—P1—C16—C21	164.2 (3)	C42—C37—C38—C39	54.9 (5)
C10—P1—C16—C21	54.5 (4)	P2—C37—C38—C39	−178.2 (3)
Pt1—P1—C16—C21	−71.0 (3)	C37—C38—C39—C40	−54.7 (6)
C1—P1—C16—C17	−72.5 (3)	C38—C39—C40—C41	55.2 (6)
C10—P1—C16—C17	177.8 (3)	C39—C40—C41—C42	−55.7 (7)
Pt1—P1—C16—C17	52.3 (3)	C38—C37—C42—C41	−55.9 (6)
C21—C16—C17—C18	−53.6 (5)	P2—C37—C42—C41	175.9 (4)
P1—C16—C17—C18	−178.3 (3)	C40—C41—C42—C37	56.4 (7)
